# Effects of L-carnitine and pentoxifylline on long-term preservation of the human sperms: An experimental study

**DOI:** 10.18502/ijrm.v22i11.17820

**Published:** 2025-01-10

**Authors:** Elham Aliabadi, Zohre Nateghian, Mohammad Hossein Nasr-Esfahani, Marziyeh Tavalaee, Tahereh Talaei-Khozani

**Affiliations:** ^1^Department of Anatomy, School of Medicine, Shiraz University of Medical Sciences, Shiraz, Iran.; ^2^Islamic Azad University of Isfahan (Khorasgan) Branch, Isfahan, Iran.; ^3^Department of Animal Biotechnology, Reproductive Biomedicine Research Center, Royan Institute for Biotechnology, ACECR, Isfahan, Iran.; ^4^Isfahan Fertility and Infertility Center, Isfahan, Iran.; ^5^Histomorphometry and Stereology Research Center, Anatomy Department, School of Medicine, Shiraz University of Medical Sciences, Shiraz, Iran.

**Keywords:** Apoptosis, Sperm motility, L-carnitine, Pentoxifylline, Semen preservation.

## Abstract

**Background:**

In infertility clinics, long-time preserving high-quality spermatozoa is a challenging problem.

**Objective:**

The present study aimed to prolong preserving of the human spermatozoa by adding pentoxifylline (PT) and L-carnitine (LC) without using high-cost freezing techniques.

**Materials and Methods:**

In this experimental study, semen samples of 26 normozoospermia men aged between 28–34 yr, were firstly prepared using the swim-up technique, and each sample was divided into the following 3 aliquots: untreated control group, the LC, and PT-treated groups. The samples were stored for up to 12 days at 4–6 C, and sperm motility was assessed. The percentages of the sperms with double-stranded DNA, apoptotic, and acrosomal interacted sperms were evaluated by sperm chromatin structure assay, AnnexinV-PI staining, and peanut agglutinin, respectively.

**Results:**

On day 7, 26.83% ± 4.26 of sperms were motile in the PT group which was significantly more than LC (6.67% ± 0.61) and control (0.83 ± 0.17) groups (p < 0.001). At day 12, while all sperms lost their motility in LC and control groups, adding PT led to 3.17% ± 0.47 sperms remaining motile (p < 0.001). Moreover, on day 12, the percent of apoptotic sperms in the PT-treated group (8% ± 0.20) was significantly lower than in LC-treated group (5.9% ± 0.28, p = 0.03). None of the additives positively affected the number of sperms with double-stranded DNA (p > 0.05). LC could also maintain acrosomal integrity over a storage time of up to 12 days.

**Conclusion:**

Despite PT's improved sperm motility, LC was more efficient in preventing apoptosis and acrosomal reactions. However, DNA was resistant to denaturation regardless of the treatments.

## 1. Introduction

In assisted reproductive technology (ART), keeping sperm for hours or days is a necessity, which has detrimental impacts on sperm quality (1). Since freezing methods are expensive and have a detrimental impact on sperms, it is essential to find a non-frozen storage medium. Based on the results of previous studies, the more viable and motile sperms were present whenever incubated at 4–6 C compared to 25 C (2).

During in vitro storage, reactive oxygen species (ROS) produced by abnormal spermatozoa can be considered as one of the reasons for ART technique failure and reduces sperm functions through lipid peroxidation (3).

Reducing antioxidant enzymes by ROS in semen is another reason for sperm death, and investigations showed that adding antioxidants improves the viability of cold-stored sperms, this can be a good way to prevent sperm damage during in vitro storage (4).

In this regard, it seems that the use of L-carnitine (LC) and pentoxifylline (PT) as antioxidants in the culture medium is useful for inhibiting sperm motility and loss of viability.

LC is one of the compounds that can improve sperm motility in living bodies in vivo and in vitro (5). In addition, it has been suggested as a therapeutic compound in improving the quality of sperm motility extracted from the testis (6). LC is a derivative of the amino acid lysine and methionine, made by the mammalian body in the diet and kidney, and is then stored in the skeletal muscles, heart, brain, and sperm (7). In men, more carnitine is found in seminal vesicles (epididymal tissue, seminal plasma, and sperm). LC seems to play a key role in sperm metabolism by providing the energy required by sperm and having an influential effect on sperm maturation and motility (8).

The results of many studies have indicated that LC (0.5–1.0 mg/ml^-1^) improves the motility and survival of human sperm, protects against DNA damage following sperm freezing, and preserves mitochondrial function in sperm stenotic samples, thereby improving sperm load and quality (3, 9).

PT is also an antioxidant that potentially stimulates sperm motility. PT stimulates sperm motility by inhibiting cyclic adenosine monophosphate (cAMP) phosphodiesterase and the beneficial effects of PT occur via the incremented cAMP levels (10). PT significantly improves sperm parameters in cases with oligoasthenoteratozoospermia (11). Although the use of PT does not increase the number of motile sperm, it improves the characteristics of sperm motility (12). The improvement of sperm motility in oligozoospermic and asthenozoospermic samples can improve the results of in vitro fertilization or intrauterine insemination (13). Oral administration of PT can be an effective treatment for improving semen and sperm parameters (14).

Considering the effects of LC and PT on sperm quality and the lack of studies on the effects of these 2 antioxidants at non-freezing temperatures, this study aimed to investigate the effects of LC and PT treatments on sperm quality incubated for various intervals at 4–6 C.

## 2. Materials and Methods

### Study design and sample size

In this experimental study, semen samples of 26 normozoospermic men who were referred to the Andrology Unit of Fertility and Infertility Center, Isfahan, Iran from September to December 2022 were collected. Samples were, firstly, prepared using the swim-up technique, rinsed twice in modified Ham's F10, resuspended in vitro fertilization medium, and finally each sample was divided into the following 3 aliquots: an untreated control group, the LC, and PT-treated groups. Those aged between 28 and 34 yr with normal morphology, sperm count, motility, and viability according to the World Health Organization guidelines criteria included in the study (15).

Men with pathological semen analysis results, varicocele, undescended tested, or a history of orchiopexy, testis surgery, or solitary testis (since this would affect the semen analysis findings) were excluded from the study.

Of this population at the confidence level of 95% and the test power of 80% as well as taking into account the results of previous studies indicating the standard deviation of motile sperm percentage in the LC and PT groups to be equal to 0.61 and 4.26, respectively, the error level resulting from the minimum difference in the mean of the 2 groups to be equal to 2.5, and the possible dropout of 10%, the sample size was estimated to be 26 cases.

### Semen preparation and grouping

After 2–7 sexual abstinence days, semen samples were collected by masturbation into sterile containers. After liquefying the semen for 30 min at 37 C, the routine semen analysis was conducted using a computer-assisted sperm analysis system (CASA, VT-Sperm Test, 2.3 model-Company of Video Test-Finland) in terms of the World Health Organization. Motile sperms were collected by swim-up and then washed twice in modified Ham's F10 (Sigma, USA, Cat No# N6908) containing 5% human serum albumin (KedrionSpA, Italy, Cat No# S-P-01595). Thereafter, the samples were aliquoted into 3 parts, the control samples were kept in modified Ham's F10 without any treatment. 2 other samples were kept in a medium supplemented with either 1.8 mM LC or 1.8 mM PT (both from Sigma, USA, Cat No# C0283 and P1784, respectively) (16). All the aliquots were incubated for up to 12 days at 4–6 C. Immediately after sampling and on days 1, 2, 5, 7, and 12 after incubation, a sample of each aliquot was taken for further tests.

### Sperm motility assessment

The total motility was determined by placing 10 μ L of sperm samples in a Mackler chamber, and then, the percentage of motile sperms were evaluated by a computer-assisted sperm analysis system.

### Sperm chromatin structure assay

To assess sperm DNA integrity and DNA fragmentation index (DFI), sperm concentration was adjusted at 2 × 10^6^ per mL in Tris-HCl, NaCl, and EDTA buffer; pH 7.4 (0.01 M Tris-HCl [Merck, Germany, Cat No# PHG0002] /0.15 M NaCl [Merck, Germany, Cat No# NIST3530], 1 mM EDTA [Sigma, USA, Cat No# E6758]). In experimental groups, 400 μ L of acid-detergent solution (0.08 N HCl, 0.15 M NaCl, and 0.1% [v:v] Triton X-100 [Sigma, USA, Cat No# T8787]) was added to 200 μ L of diluted samples in Tris-HCl, NaCl and EDTA buffer, and after 30 sec, they were mixed with 1200 μ L of staining buffer. The staining buffer contained 6 µg/mL acridin orange (Sigma, USA, Cat No# 235474) in 0.1 M citric acid (pH 6.0, Sigma, USA, Cat No# 251275/0.2 M), Na_2_PO_4_ (Merck, Germany, Cat No# 105108), 1 mM EDTA, and 0.15 M NaCl. In the control group, 1200 μ L of staining buffer was added to 200 μ L of the diluted sample without adding an acid-detergent solution. Finally, the percentage of reacted sperms with acridine orange was determined by flow cytometry (Becton Dickinson) and DFI was obtained by following formula (17).

The DFI was calculated as follows: 


$$\text{DIF}=\frac{\text{The}\text{mean}\text{percentage}\text{od}\text{sperm}\text{related}\text{to}\text{FL3}\text{channel}\text{(denaturated}\text{sperms)}}{\text{The}\text{mean}\text{percentage}\text{of}\text{sperms}\text{related}\text{to}\text{FL1+FL3}\text{(total}\text{sperms)}}$$

### Apoptosis assessment

Apoptosis were evaluated by phosphatidylserine translocation. According to the instructions provided by the AnnexinV-PI kit (Biolegend Products), 1 × 10^6^ sperms were mixed with 200 μ L of cell staining buffer and centrifuged for 5 min at 2000 rpm. The pellet resuspended in 200 μ L of Annexin binding buffer. Thereafter, 5 µL of Annexin-V (An) and 10 μ L of propidium iodide (PI) were added and incubated for 15 min in the dark. As technical control, 100 μ L of the diluted sperms was added to Annexin binding buffer without adding An or PI. Finally, 300 μ L of Annexin binding buffer was added to each sample and examined by a fluorescence-activated cell sorter caliber flow cytometer (Becton Dickinson) equipped with Cell Quest (version6–2008) software. Dot plots showed 4 subpopulations of spermatozoa in 4 quadrants; live intact spermatozoa, (An-PI-, lower left quadrant); the spermatozoa (An+PI- lower right quadrant) in early apoptotic stage; dead spermatozoa, (An-PI+, upper left quadrant); and dead spermatozoa (An + PI+, upper right quadrant), in late apoptotic stage (17).

### Acrosomal reaction assessment

Fluorescein isothiocyanate (FITC)-conjugated peanut agglutinin (PNA, Sigma, USA, Cat No# L7381) was used to stain the sperm acrosomal content and evaluate the acrosomal reaction (18). At first, the control, LC-, and PT-treated groups were washed with 800 mL phosphate-buffered saline (PBS), centrifuged for 10 min at 1200 rpm, and fixed with 2% paraformaldehyde for 30 min at 4 C. Thereafter, the aliquots were centrifuged and the pellets were resuspended in PBS. FITC-conjugated PNA was added to each aliquot at the dilution of 10 µg/mL for 2 hr in darkness at 37 C and a humidified environment. After rinsing with PBS, the fluorescence-1 channel of flow cytometry was used to assess the frequencies of the spermatozoa stained with FITC-conjugated lectin. Flowjo software was used to analyze the obtained data.

### Ethical Considerations

The experiments in this study were approved by the Ethics Committees of Shiraz University of Medical Sciences, Shiraz, Iran (Code: IR.SUMS.REC.1399.769) and Isfahan Royan Institute, Isfahan, Iran (Code: IR.ACECR.ROYAN.REC.1400.017). Informed written consent was obtained from all participants.

### Statistical Analysis

All the data were analyzed by Statistical Package for Social Studies (SPSS software V.25, SPSS Inc.). The data were stated as mean ± standard deviation. All the variables were examined using the Kolmogorov-Smirnov test in terms of normal distribution. The results were compared with a one-way analysis of variance (ANOVA) and followed by the least significant difference test. P < 0.05 were statistically significant.

## 3. Results

### Sperm total motility assessment

**Figure 1:**
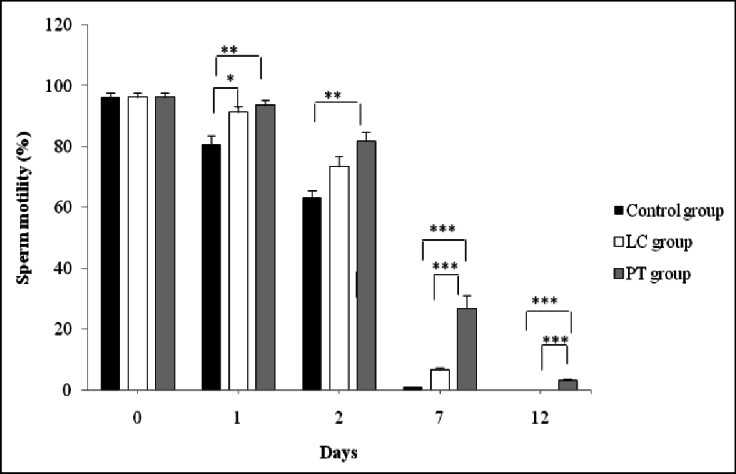
Trend of changes in total motility of sperm in control, LC, and PT groups on different days of storage. PT improved the frequency of the total motile sperm compared with LC and control. PT: Pentoxifylline, LC: L-carnitine. *P < 0.05, **P < 0.01, ***P < 0.001.

The percentage of total sperm motility (progressive and non-progressive sperms) on the 1 ${\;}^{\text{st}}$ day of incubation in the control group with the mean of 80.83 ± 2.96 was significantly lower than that of PT and LC groups with the means of 93.67 ± 1.84, 3.17 ± 0.47, and 91.33 ± 1.85, respectively (p = 0.01). However, the LC and PT groups did not have significant differences with each other (p > 0.05). On the 7 ${\;}^{\text{th}}$ and 12 ${\;}^{\text{th}}$ day, PT administration, as compared to LC, showed a better preventive effect on the loss of sperm motility (p < 0.001). On the 12 ${\;}^{\text{th}}$ day of incubation, although there were no motile sperm in the control and LC groups, the percentage of motile sperm in the PT group was reported to have the mean of 3.17 ± 0.47% (p < 0.001) (Figure 1).

### Immotile sperms assessment

**Figure 2:**
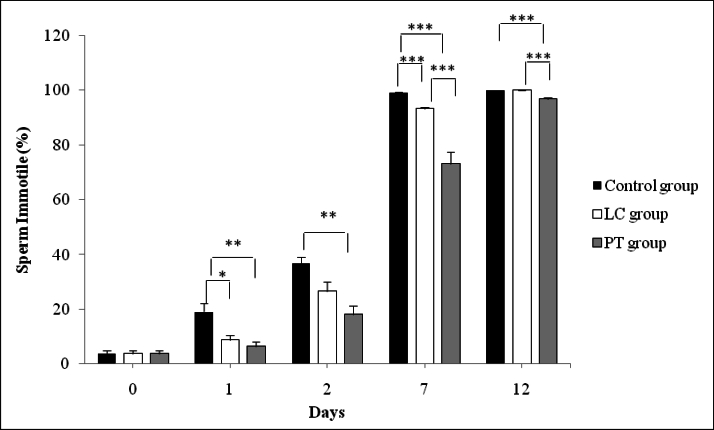
Trend of changes in immotile sperms in control, LC, and PT groups on different days of storage. PT: Pentoxifylline, LC: L-carnitine. *P < 0.05, **P < 0.01, ***P < 0.001.

A significant difference was observed between the control and PT groups in the percentage of immotile sperm from the 1 ${\;}^{\text{st}}$ to the 12 ${\;}^{\text{th}}$ days (p < 0.001). The percentage of immotile sperm until the 12 ${\;}^{\text{th}}$ day was higher in the control group. Moreover, the percentage of immotile sperm the LC and control groups was also significantly different from the 1 ${\;}^{\text{st}}$ day to the 5 ${\;}^{\text{th}}$ day (p < 0.001), such that the percentage of immotile sperm in the control group was higher than its percentage in the LC group. The percentage of immotile sperm in the PT group with the means of 73.17 ± 4.26 and 96.83 ± 0.48 on days 7 and 12, respectively was significantly lower than its percentage in the LC group with the means of 93.33 ± 0.61 and 100.00 ± 0.00 on days 7 and 12, respectively (p < 0.001). However, all sperm (100%) were immobile in the control and LC groups on the 12 ${\;}^{\text{th}}$ day (Figure 2).

### Sperm DNA integrity assessment 

**Figure 3:**
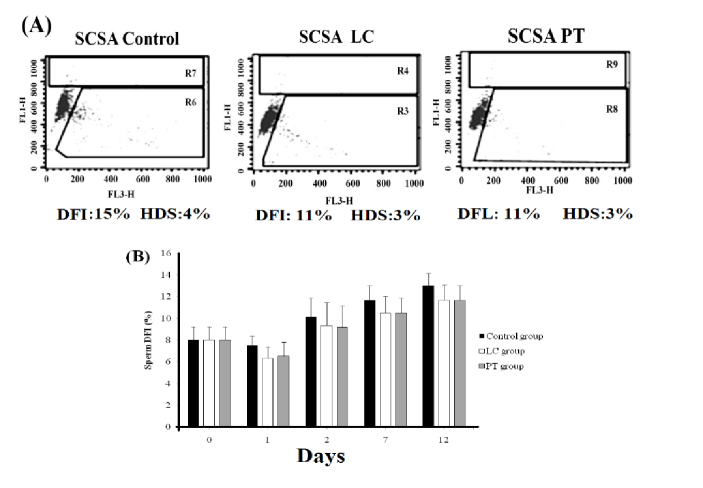
A) A representative sample of dot blot of SCSA in control, LC, and PT groups over 12 days of storage. B) Trend of changes in sperm DNA fracture index in control, LC, and PT groups on different days of storage. The DNA fracture index was similar regardless of the treatment at each point of time. LC: L-carnitine, PT: Pentoxifylline, SCSA: Sperm chromatin structure assay, DFI: DNA fragmentation index, HDS: High DNA stainability.

DFI increased gradually from day 0 to day 12. However, neither PT nor LC exposure could prevent DNA denaturation (Figure 3).

### Apoptosis assessment

**Figure 4:**
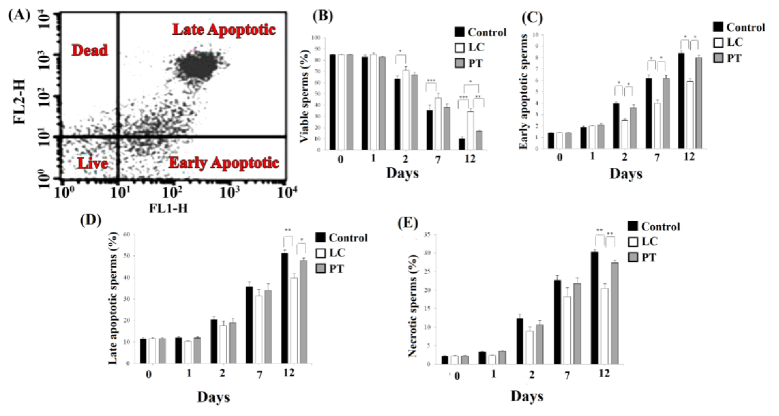
A) A representative dot blot sample shows the live sperms (lower left quadrant), early apoptotic (lower right quadrant), late apoptotic (upper right quadrant), and necrotic (upper left quadrant) regions. B) The trend of changes in the frequency of the viable, C) Early apoptotic, D) Late apoptotic, and E) Necrotic sperm in control, LC, and PT groups on different days of storage. PT: Pentoxifylline, LC: L-carnitine. *P < 0.05, **P < 0.01, ***P < 0.001.

The frequency of different subpopulations of annexin V-PI-positive spermatozoa is summarized in figure 4A. Although the exposure of the sperms with LC led to an increase in the live sperms compared to matched time control groups from days 2–12 (p = 0.04, p < 0.001, and p < 0.001, for days 2, 7, and 12, respectively), it was statistically similar to corresponding PT groups. However, at day 12, the number of live sperms (viability sperm without apoptosis) in LC group with a mean of 34.10 ± 2.94 was significantly higher than its number in PT group with a mean of 16.80 ± 0.74 as well (p = 0.01, Figure 4B).

On days 2, 7, and 12, the percentage of early apoptotic sperms in the LC group with the means of 2.50 ± 0.13, 4.00 ± 0.28, and 5.90 ± 0.28, were significantly lower than their percentage in the PT group with the means of 3.60 ± 0.27, 6.20 ± 0.26, and 8.00 ± 0.20, and their percentage in the control group with the means of 4.00 ± 0.08, 6.20 ± 0.30, and 8.40 ± 0.20, respectively (p < 0.05). However, no significant difference was observed between PT and control groups (p > 0.05) (Figure 4C).

Moreover, on the 12 ${\;}^{\text{th}}$ day, the percentage of late apoptotic and necrosis sperms in the LC group with the means of 39.6 ± 2.24 and 20.40 ± 1.40, respectively was significantly lower than their percentage in the PT group with the means of 47.80 ± 1.29 and 27.40 ± 0.75, and their percentage in the control group with the means of 51.20 ± 1.66 and 30.20 ± 0.78, respectively (p = 0.01) (Figure 4D, E).

### Acrosomal reaction assessment

**Figure 5:**
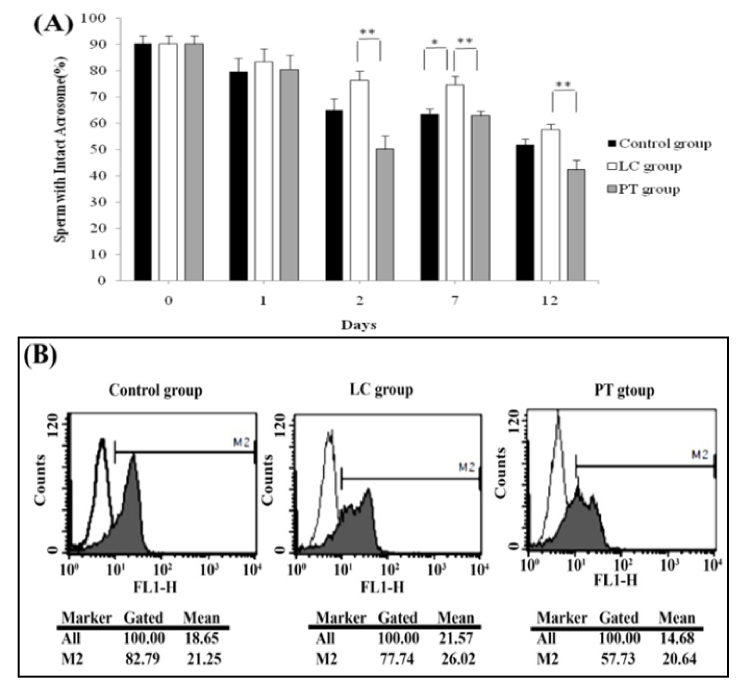
A) The trend of changes in the percentage of healthy sperms with intact acrosome in control, LC, and PT groups on different days of storage. B) The flow cytometry of a representative sample of different groups on 12 days. PT: Pentoxifylline, LC: L-carnitine. *P < 0.05, **P < 0.01.

Healthy sperms with intact acrosome were stained with PNA and acrosomal reacted sperms failed to stain with the lectin. LC exposure kept the acrosomal integrity compared to control aliquots in just 7 days (p = 0.01). Adding PT to the aliquots had no significant impact on the acrosomal integrity of the sperms on all days. It seems that LC was more efficient in keeping acrosomal integrity than PT, as in most points of time, PT even led to no significant decrease in the percentages of sperms with intact acrosome (Figure 5A, B).

## 4. Discussion

According to the results of the present study, the PT-supplemented medium, as compared to LC and control media, was significantly more effective in improving sperm motility at 4–6 C during 12 days. In detail, on the 12 ${\;}^{\text{th}}$ day, no motile sperm were observed in the control and LC groups (100% immotile sperm); however, an average of 3% motile sperm remained in the PT group. In our previous study, adding PT leads to a reduction in sperm motility after 180 min of incubation at room temperature (3). It seems that reducing temperature is effective in reducing PT toxicity, while it can preserve sperm motility. In contrast to our study, incubation of stallion sperms with PT at 4 C for 24 hr, reduced motility; however, the doses used in that study were different (19). Cooling goat sperms in the present PT also improved sperm motility (20). Therefore, the long-term effects of PT seem to be species-specific or dose dependent.

Some mechanisms have been suggested for increasing sperm motility by incubating in PT. A previous report suggested that PT maybe an inhibitor of the cAMP phosphodiesterase enzyme, which increases the sperms motility by elevating intracellular cAMP concentrations (10). In another study, PT was detected to increase the level of nitric oxide produced by the sperms; thereby improving its motility (21).

In addition, the results of the present study showed that PT, compared to LC, only had an effective role in improving sperm motility, but it did not have a significant effect on other sperm quality parameters such as DFI, early and late apoptotic, and necrosis sperms. In this regard, the percentage of early apoptotic sperms in LC group was significantly less than their percentage in the PT and control groups on days 2, 7, and 12. However, the PT and control groups did not have significant differences with each other. Moreover, on the 12 ${\;}^{\text{th}}$ day, the percentage of late apoptotic and necrosis sperms in LC group was significantly lower than their percentage in PT and control groups. In fact, LC treatment prevents sperm apoptosis and necrosis and improves sperm viability during 12 days of incubation.

In contrast to our findings, Nazari and co-workers found that administration of 3 and 6 mM PT to goat sperms in the chilling state improved the sperm quality (20), which may be due to higher PT concentration in the cooling medium; however, prolonged incubation is toxic. Storage of the buck's sperm at 4 C at the present of 5 mM LC for 48 hr increased the percentage of motile sperms (22). Supplementation of the preservation media with 1 and 2 mM LC for 24 and 48 hr at 5 C also improved ram sperm motility (23).

Experimental evidence showed that antioxidant supplements can usefully prevent the formation of ROS and improve the quality of frozen human sperm after thawing. According to the majority of human clinical trials, in patients with asthenozoospermia, the concentration of LC in the seminal plasma is much lower than that of the normozoospermic control group, and treatment with LC and acetyl-L-carnitine may lead to the optimization of sperm motility factors in asthenozoospermic or oligoasthenospermia men (5). In addition to oxidative stress, the increase of apoptotic sperms is another factor causing damage in the freezing process, and LC also has antiapoptotic activity (24). In our study, 2 points are important, firstly, a non-freezing medium was used and secondly, antioxidants such as LC and PT were used to maintain the sperm quality parameters.

In line with our study, a previous investigation suggested that LC increases sperm viability by boosting glucose uptake due to its antioxidant properties (25). LC may exert its effects through different mechanisms. L-acetyl carnitine protects the stem cells from apoptosis through inducing autophagy when the cells are deprived of sufficient nutrients (26). Also, oral administration of LC to rats has been reported to protect genotoxicity by enhancing autophagy (27). Autophagy in sperm plays an important role in acrosome reaction (28). Besides, supplementation freezing medium with LC prevented sperm apoptosis, improved sperm viability and membrane integrity, and elevated the total antioxidant capacity (29). On the other hand, in vitro incubation of sperms from asthenoteratozoospermia participants for 6 hr diminished DNA fragmentation and increased mitochondrial membrane potential (24). To the best of our knowledge, this is the first study that shows the preventive long-term effects of PT and LC on sperm apoptosis in a nonfrozen state.

The data from the current study showed that the percentage of healthy spermatozoa with acrosomal integrity was significantly higher in LC-treated aliquots after a 12-day storage than in both control and PT groups. This was consistent with the results of the study by Aliabadi et al. who evaluated acrosomal reactions in freeze-thawed human ejaculated spermatozoa treated with PT and LC. Based on their results LC protected the acrosomal and plasma membrane integrity (18).

Hence, LC may be considered the better option to maintain sperm integrity compared to PT. LC supplementation has been shown to improve the membrane integrity of buck sperms preserved at 4 C (22). Also, cooling ram sperms at 5 C in the presence of LC improved acrosomal integrity in a dose-dependent manner (23). Sperm chromatin structure assay showed that DNA integrity was not changed by a long-term storing of spermatozoa with LC and PT. This result may be attributed to the extreme compaction of sperm chromatin, and as a result, the degradation of the sperm nucleus may be delayed, since the plasma membrane is more susceptible to damage by the chemicals such as ROS in the culture medium. This DNA impaction protects sperms, from numerous mechanical and chemical insults during transit in both the female and male genital tracts.

It should be noted that the tolerance of the various species to cooling shock is different. For example, the tolerance threshold of stallion sperms to chilling is less than the other species. It may relate to the different cholesterol content than in the membrane of the sperms from different species (30). Therefore, the controversial results may be attributed to the species.

Although this study showed the beneficial effects of 2 antioxidants, that is, LC and PT, in preserving human sperm for 12 days, it seems that the small sample size, not using more tests including other methods of sperm chromatin evaluation, such as single-cell electrophoresis (Comet test) or the marking method instead of DNA breaks (Tunel test) as well as nonstorage of more than 12 days can be considered as some of the limitations of this study. Therefore, further investigations in this respect with a larger sample size, longer follow-up days, and also using other sperm chromatin evaluation methods can better investigate sperm parameters and the integrity of its chromatin over a longer period of time. Therefore, the results can be generalized to the population with more confidence.

## 5. Conclusion

It is concluded that the DNA of human spermatozoa resisted denaturation even in long-term preservation. PT could be a mor eeffective choice for the improvement of total sperm motility, whereas, LC could be a good choice for protecting the human sperms against apoptosis and spontaneously acrosomal reaction after 12 days of storage at 4–6 C. Our finding suggests a way for long-term preservation of good quality sperms in fertility clinics.

##  Data Availability

Data supporting the findings of this study are available upon reasonable request from the corresponding author.

##  Author Contributions

Z. Nateghian and MH. Nasr-Esfahani had complete access to all data used in the study and took responsibility for both the integrity of the data and the accuracy of the data analysis, including the conceptualization and design of the study. E. Aliabadi, M. Tavalaee, and T. Talaei-Khozani were responsible for the acquisition, analysis, and interpretation of the data. Z. Nateghian and MH. Nasr-Esfahani drafted the manuscript and conducted statistical analysis. All authors contributed to the critical revision of the manuscript for significant intellectual content and approved the final manuscript and took responsibility for the integrity of the data.

Elham Aliabadi and Zohre Nateghian are both first authors due to same involvement in conceptualization, data analyses and writing the draft.

##  Acknowledgments

This work was supported by deputy research and technology of Shiraz University of Medical Sciences, Shiraz, Iran (grant number: 21160), and the Royan Institute, Isfahan, Iran. AI was not used to prepare the manuscript in any way (translation, revision, grammar check, etc.).

##  Conflict of Interest

The authors declare that there is no conflict of interest.

## References

[bib1] Akbarzadeh-Jahromi M, Jafari F, Parsanezhad ME, Alaee S (2022). Evaluation of supplementation of cryopreservation medium with gallic acid as an antioxidant in quality of post-thaw human spermatozoa. Andrologia.

[bib2] Nateghian Z, Nasr-Esfahani MH, Talaei-Khozani T, Tavalaee M, Aliabadi E (2023). L-carnitine and pentoxifylline supplementation improves sperm viability and motility at low temperature. Int J Fertil Steril.

[bib3] Chianese R, Pierantoni R (2021). Mitochondrial reactive oxygen species (ROS) production alters sperm quality. Antioxidants.

[bib4] Ibáñez-Arancibia E, Farías JG, Valdebenito I (2021). Use of antioxidants and time of cold storage: Effects over viability parameters and enzymatic levels in semen of rainbow trout (Oncorhynchus mykiss, Walbaum, 1792). Braz J Biol.

[bib5] Mateus FG, Moreira S, Martins AD, Oliveira PF, Alves MG, Pereira MD

[bib6] Kooshesh L, Nateghian Z, Aliabadi E (2023). Evaluation of L-carnitine potential in improvement of male fertility. J Reprod Infertil.

[bib7] Prakash N, Ghosal S, Maity M (2023). A review on therapeutic effects of L-carnitine: An update. J Adv Zool.

[bib8] Zhang X, Cui Y, Dong L, Sun M, Zhang Y (2020). The efficacy of combined L‐carnitine and L‐acetyl carnitine in men with idiopathic oligoasthenoteratozoospermia: A systematic review and meta‐analysis. Andrologia.

[bib9] Hufana-Duran D, Duran PG, Monson R, Parrish J (2018). Motility and membrane integrity of ejaculated bovine spermatozoa extended and cryopreserved in L-carnitineTris-egg yolk extender. J ISSAAS.

[bib10] Nazari M, Daghigh Kia H, Najafi A (2022). [Effect of pentoxifylline antioxidant supplementation on improvement of sperm motility parameters in non-breeding season]. Iran J Anim Sci Res.

[bib11] Dadgar Z, Shariatzadeh SM, Mehranjani MS, Kheirolahi A (2022). The therapeutic effect of co-administration of pentoxifylline and zinc in men with idiopathic infertility. Iran J Med Sci.

[bib12] Lu Y, Su H, Zhang J, Wang Y, Li H (2022). Treatment of poor sperm quality and erectile dysfunction with oral pentoxifylline: A systematic review. Front Pharmacol.

[bib13] Mahaldashtian M, Khalili MA, Nottola SA, Woodward B, Macchiarelli G, Miglietta S

[bib14] Safarinejad MR (2011). Effect of pentoxifylline on semen parameters, reproductive hormones, and seminal plasma antioxidant capacity in men with idiopathic infertility: A randomized double-blind placebo-controlled study. Int Urol Nephrol.

[bib15] Björndahl L, Brown JK, Other editorial board members of the WHO Laboratory Manual for the Examination and Processing of Human Semen (2022). The sixth edition of the WHO Laboratory Manual for the Examination and Processing of Human Semen: Ensuring quality and standardization in basic examination of human ejaculates. Fertil Steril.

[bib16] Aliabadi E, Karimi F, Talaei-Khozani T (2013). Effects of L-carnitine and pentoxifylline on carbohydrate distribution of mouse testicular sperm membrane. Iran J Med Sci.

[bib17] Evenson DP (2016). The sperm chromatin structure assay (SCSA®) and other sperm DNA fragmentation tests for evaluation of sperm nuclear DNA integrity as related to fertility. Anim Reprod Sci.

[bib18] Aliabadi E, Jahanshahi S, Talaei‐Khozani T, Banaei M (2018). Comparison and evaluation of capacitation and acrosomal reaction in freeze‐thawed human ejaculated spermatozoa treated with L‐carnitine and pentoxifylline. Andrologia.

[bib19] Rossi M, Gonzalez-Castro R, Falomo ME (2020). Effect of caffeine and pentoxifylline added before or after cooling on sperm characteristics of stallion sperm. J Equine Vet Sci.

[bib20] Nazari P, Farshad A, Hosseini Y (2022). Protective effects of trehalose and pentoxifylline on goat sperm exposed to chilling-freezing process. Biopreserv Biobank.

[bib21] Banihani SA, Abu‐Alhayjaa RF, Amarin ZO, Alzoubi KH (2018). Pentoxifylline increases the level of nitric oxide produced by human spermatozoa. Andrologia.

[bib22] Heydari M, Qasemi-Panahi B, Moghaddam Gh, Daghigh-Kia H, Masoudi R (2021). Conservation of buck’s spermatozoa during cooling storage period through cooling medium supplementation with L-carnitine. Arch Razi Inst.

[bib23] Galarza DA, López‐Sebastián A, Santiago‐Moreno J (2020). Supplementing a skimmed milk-egg yolk‐based extender with L‐carnitine helps maintain the motility, membrane integrity and fertilizing capacity of chilled ram sperm. Reprod Domest Anim.

[bib24] Naderi Noreini S, Malmir M, Ghafarizadeh A, Faraji T, Bayat R (2021). Protective effect of L‐carnitine on apoptosis, DNA fragmentation, membrane integrity and lipid peroxidation of spermatozoa in the asthenoteratospermic men. Andrologia.

[bib25] Abd‐Elrazek AM, Ahmed‐Farid OA (2018). Protective effect of L‐carnitine and L‐arginine against busulfan‐induced oligospermia in adult rat. Andrologia.

[bib26] Pan T, Qian Y, Li T, Zhang Z, He Y, Wang J, et al (2022). Acetyl L-carnitine protects adipose-derived stem cells against serum-starvation: Regulation on the network composed of reactive oxygen species, autophagy, apoptosis and senescence. Cytotechnology.

[bib27] Khedr NF, Werida RH (2022). L-carnitine modulates autophagy, oxidative stress and inflammation in trazodone induced testicular toxicity. Life Sci.

[bib28] Wang M, Zeng L, Su P, Ma L, Zhang M, Zhang YZ (2022). Autophagy: A multifaceted player in the fate of sperm. Hum Reprod Update.

[bib29] Abdelnour SA, Hassan MA, El‐Ratel IT, Essawi WM, El‐Raghi AA, Lu Y, et al (2022). Effect of addition of L‐carnitine to cryopreservation extender on rabbit post‐thaw semen parameters, antioxidant capacity, mitochondrial function, apoptosis and ultrastructure changes. Reprod Domest Anim.

[bib30] Gibb Z, Aitken RJ (2016). The impact of sperm metabolism during in vitro storage: The stallion as a model. Biomed Res Int.

